# Primary Cardiac Sarcoma: Clinical Characteristics and Prognostic Factors over the Past 2 Decades

**DOI:** 10.3390/diseases11020074

**Published:** 2023-05-14

**Authors:** Ayrton Bangolo, Pierre Fwelo, Kritika M. Iyer, Sarah Klinger, Lorena Tavares, Shraboni Dey, Angel Ann Chacko, Myat Hein, Samyukta Gudena, Gbenga Lawal, Barath P. Sivasubramanian, Zekordavar Rimba, Kinjal Hirpara, Merajunnissa Merajunnissa, Swathi Veliginti, Georgemar Arana, Dily T. Sathyarajan, Sachin Singh, Tanvi Shetty, Kshitij Bhardwaj, Sayed Hashemy, Roberto L. Duran, Sung H. Kim, Candice M. Hipolito, Kibo Yoon, Vrusha Patel, Aseel Alshimari, Pugazhendi Inban, Saaniya Yasmeen, Krushika Devanaboyina, Gulshan Kumar, Saran Preet, Mishgan Akhtar, Ayanleh Abdi, Navya Nalajala, Syed F. M. Rizvi, Bhavna Gupta, Simcha Weissman

**Affiliations:** 1 Department of Medicine, Hackensack Meridian Health/Palisades Medical Center, North Bergen, NJ 07047, USA; 2 Department of Epidemiology, Human Genetics, and Environmental Sciences, UTHealth School of Public Health, Houston, TX 77030, USA; 3 Department of Hematology and Oncology, Hackensack Meridian Health/Palisades Medical Center, North Bergen, NJ 07047, USA

**Keywords:** primary cardiac sarcoma, SEER database, prognostic factors, clinical characteristics

## Abstract

Background: Primary cardiac sarcomas (PCS) are extremely rare malignant tumors involving the heart. Only isolated case reports have been described in the literature over different periods of time. This pathology has been associated with a dismal prognosis and given its rarity; treatment options are very limited. Furthermore, there are contrasting data about the effectiveness of current treatment modalities in improving the survival of patients with PCS, including surgical resection which is the mainstay of therapy. There is a paucity of data on the epidemiological characteristics of PCS. This study has the objective of investigating the epidemiologic characteristics, survival outcomes, and independent prognostic factors of PCS. Methods: A total of 362 patients were ultimately registered in our study from the Surveillance, Epidemiology, and End Results (SEER) database. The study period was from 2000 to 2017. Demographics such as clinical characteristics, overall mortality (OM), and PCS-specific mortality (CSM) were taken into account. A *p* value of <0.1 in the univariate analysis leads to the incorporation of the variable into multivariate analysis adjusting for covariates. Adverse prognostic factors were represented by a Hazard Ratio (HR) greater than one. The five-year survival analysis was carried out using the Kaplan–Meier method and the log-rank test was used to compare survival curves. Results: Crude analysis revealed a high OM in age 80+ (HR = 5.958, 95% CI 3.357–10.575, *p* < 0.001), followed by age 60–79 (HR = 1.429, 95% CI 1.028–1.986, *p* = 0.033); and PCS with distant metastases (HR = 1.888, 95% CI 1.389–2.566, *p* < 0.001). Patients that underwent surgical resection of the primary tumor and patients with malignant fibrous histiocytomas (HR = 0.657, 95% CI 0.455–0.95, *p* = 0.025) had a better OM (HR = 0.606, 95% CI 0.465–0.791, *p* < 0.001). The highest cancer-specific mortality was observed in age 80+ (HR = 5.037, 95% CI 2.606–9.736, *p* < 0.001) and patients with distant metastases (HR = 1.953, 95% CI 1.396–2.733, *p* < 0.001). Patients with malignant fibrous histiocytomas (HR = 0.572, 95% CI 0.378–0.865, *p* = 0.008) and those who underwent surgery (HR = 0.581, 95% CI 0.436–0.774, *p* < 0.001) had a lower CSM. Patients in the age range 80+ (HR = 13.261, 95% CI 5.839–30.119, *p* < 0.001) and advanced disease with distant metastases (HR = 2.013, 95% CI 1.355–2.99, *p* = 0.001) were found to have a higher OM in the multivariate analyses adjusting for covariates). Lower OM was found in patients with rhabdomyosarcoma (HR = 0.364, 95% CI 0.154–0.86, *p* = 0.021) and widowed patients (HR = 0.506, 95% CI 0.263–0.977, *p* = 0.042). Multivariate cox proportional hazard regression analyses of CSM also revealed higher mortality of the same groups, and lower mortality in patients with Rhabdomyosarcoma. Conclusion: In this United States population-based retrospective cohort study using the SEER database, we found that cardiac rhabdomyosarcoma was associated with the lowest CSM and OM. Furthermore, as expected, age and advanced disease at diagnosis were independent factors predicting poor prognosis. Surgical resection of the primary tumor showed lower CSM and OM in the crude analysis but when adjusted for covariates in the multivariate analysis, it did not significantly impact the overall mortality or the cancer-specific mortality. These findings allow for treating clinicians to recognize patients that should be referred to palliative/hospice care at the time of diagnosis and avoid any surgical interventions as they did not show any differences in mortality. Surgical resection, adjuvant chemotherapy, and/or radiation in patients with poor prognoses should be reserved as palliative measures rather than an attempt to cure the disease.

## 1. Introduction

Primary cardiac sarcomas (PCS) are the most common malignancies affecting the heart; however, overall, only 15 percent of cardiac neoplasms are malignant [1]. PCS remains a rare entity in the literature that has mainly been reported by means of case reports [2,3,4,5,6]. Almost exclusively, PCS does not arise from benign neoplasms but are believed to arise de novo [7]. 

Signs and symptoms associated with PCS mostly depend on the location of the tumor [8]. PCS involving the left atrium can present with symptoms of mitral regurgitation as they tend to grow into the left atrial lumen and cause mitral insufficiency symptoms such as dyspnea, orthopnea, pulmonary edema, and hemoptysis. Some patients can present with thrombi in their general circulation, including neurologic thrombi [9]. PCS in the right atrium can present with symptoms of tricuspid stenosis and with thrombi into the pulmonary circulation causing pulmonary emboli [9]. PCS in the left ventricle may present with rhythm abnormalities and conduction aberrations as they may be intramural. Those that are intracavitary can present with systemic emboli and outflow obstruction [9]. 

Echocardiogram is the initial testing modality to assess a PCS. Location and sometimes the type of cardiac tumors can be suggested by the echocardiogram. Detailed anatomic images and information on the chemical microenvironment within the tumor can be provided by cardiac Magnetic Resonance Imaging (CMRI) and if not available, cardiac Computed tomography can be used [10]. A coronary angiogram should be performed to map blood vessels, supplying tumors that will then help to decide on the excision technique that will be most likely to be successful. 

Cardiac tumors that are resectable on imaging are managed by complete open excision, as the tumor will be biopsied after excision. This will also help prevent complications of cardiac biopsy such as embolization [8]. For unresectable or diffuse tumors based on imaging, if the benefit outweighs the risk, a percutaneous cardiac biopsy can be performed with risks of cardiac perforation. Right-sided tumors can benefit from the intracardiac echocardiographic-guided biopsy with a greater level of precision, therefore, lower the risk of cardiac perforation [11]. 

PCS is known to proliferate rapidly and be locally invasive by infiltrating the myocardium and obstructing cardiac blood flow. Complete resection is the treatment of choice, but the disease has a high recurrence rate and a high mortality rate with a median survival of 6 to 12 months [3]. Neoadjuvant or adjuvant chemotherapy has been inconsistently used with no proven benefits [3]. 

Studies addressing the prognostic factors associated with PCS are scarce. One of the largest studies on the subject was carried out by Yin and colleagues. The study looked at patients with PCS over a period of 42 years [12]. Nevertheless, adequate power studies addressing baseline epidemiology characteristics and independent prognostic factors of patients with PCS over the past two decades alone remain pauce. 

To fill in the gap in the literature, using a large United States (US) population-based dataset, we aimed to evaluate the overall clinical characteristics as well as independent prognostic factors among patients diagnosed with PCS. 

## 2. Materials and Methods

### 2.1. Study Design and Data Source 

We performed a population-based retrospective cohort study of patients with PCS, retrieving data from standardized electronic health record information over a period from 2000 and 2017, and using the SEER research plus data, 18 registries, and November 2020 submission database (http://www.seer.cancer.gov accessed on 27 July 2021). The SEER database is sponsored by the United States National Cancer Institute (US NCI) and has information on nearly 28% of the U.S. population [13]. The SEER 18 database is is a publicly available dataset that provides data such as cancer incidence, patients’ clinicopathological features, and time-to-event data. 

### 2.2. Data Selection 

#### 2.2.1. Inclusion Criteria

All patients with PCS diagnosed from 2000 to 2017 were selected in our cohort based on (1) Primary site [c38.0] and (2) histological type [ICD-O-3: 8800–8805,8810,8811,8815,8825,8830,8840,8850,8852,8855,8890,8891,8895,8896,8900–8902,8910,9040,9041,9043,9120,9130,9180,9240] [9]. The above-mentioned ICD-9, ICD-10, and/or ICD-0–3 codes were used to extract data regarding these patients from the SEER database. 

#### 2.2.2. Exclusion Criteria

We excluded patients with an unknown age at diagnosis, race, stage of the PCS, unknown histologic type, or patients diagnosed during autopsies.

### 2.3. Study Variables

#### 2.3.1. Main Exposure

All the variables included in this cohort except year of diagnosis were used as main predictors of prognosis. The variables are as follows: Age at diagnosis, gender, race/ethnicity (non-Hispanic white, non-Hispanic black, Hispanic, and others), histological type, stage at diagnosis (localized, regional, and distant), geographic residential area, yearly income, marital status, year of diagnosis, surgery, and radiation as well as chemotherapy.

#### 2.3.2. Outcomes

Overall mortality (OM) was defined as death of any causes at the end of the study, whereas Cancer-specific mortality (CSM) was defined as death related to complications of PCS at the end of the study. 

#### 2.3.3. Survival Months

Reported date of last follow up by 31 December 2017, and date of death as reported in the SEER registry were used to calculate survival time starting from the date of diagnosis in terms of OM. In terms of cancer-specific mortality, the reported date of last follow up, and the date of cancer-associated death was used to calculate CSM, starting from the date of PCS diagnosis.

#### 2.3.4. Sociodemographic and Tumor Characteristics

Baseline characteristics obtained in our cohort are as follows: Age at diagnosis, gender, race/ethnicity (non-Hispanic white, non-Hispanic black, Hispanic, and others), year of diagnosis, histological type, stage at diagnosis (localized, regional, and distant), geographic residential area, yearly income, marital status, year of diagnosis, surgery, radiation, and chemotherapy. Histologic subtypes included are as follows: Leiomyosarcoma/spindle cell sarcoma, angiosarcoma, rhabdomyosarcoma, malignant fibrous histiocytomas, synovial sarcomas, osteosarcoma/Chondrosarcoma and Sarcoma, and NOS. There were not enough cases of liposarcoma in the database; thus, these patients were excluded. “Sarcoma, NOS” indicates no tumor subtype in patient records.

### 2.4. Statistical Analysis

The fact that Hazard rates are proportional over time constitutes the basis of Cox proportional hazard regression model. In order to be able to determine independent prognostic factors influencing the OM and CSM, variables that had a *p* value of less than 0.1 in the univariate analysis were incorporated in the multivariate analysis adjusting for covariates. Deleterious prognostic factors are associated with a hazard ratio (HR) of more than one. The threshold of statistical significance was defined by a *p* value of <0.05, with a confidence interval of 95%. The five-year survival analyses of all the variables were carried out using the Kaplan–Meier method and the log-rank test was used to compare survival curves for overall survival (OS) and cancer-specific survival (CSS). The software used to carry out the statistical test was STATA 17, and all the tests were two-sided.

## 3. Results

We ultimately enrolled a total of 362 patients with a primary diagnosis of PCS. Table 1 summarizes the baseline characteristics of patients included in our cohort. Patients with higher revenue and yearly income $75,000+ and those living in populated metropolitans (1 million people) were more likely to be diagnosed. Male patients (50.83%) are slightly more represented than their female (49.17%) counterparts. The other two well-represented groups were age range 40–59 (37.85%) and non-Hispanic whites (58.84%). The most encountered histologic subtype was angiosarcoma (42.51%), which represented nearly half the cases. The majority of diagnoses were made at the advanced disease stage with distant metastases (38.67%). Married patients constituted the majority of the study (54.42%), followed by single patients (26.80%). Most patients underwent surgical resection of the primary tumor (65.47%). There was a steady number of new cases from 2000 to 2017 with an average of 20 new cases per year. 

A crude analysis of factors associated with OM and CSM among US patients between 2000 and 2017 is demonstrated in Table 2. Age 80+ (HR = 5.958, 95% CI 3.357–10.575, *p* < 0.001), followed by age 60–79 (HR = 1.429, 95% CI 1.028–1.986, *p* = 0.033); and PCS with distant metastases (HR = 1.888, 95% CI 1.389–2.566, *p* < 0.001) have the highest OM. Patients that underwent surgical resection of the primary tumor (HR = 0.606, 95% CI 0.465–0.791, *p* < 0.001) and patients with malignant fibrous histiocytomas (HR = 0.657, 95% CI 0.455–0.95, 169 *p* = 0.025) had a better OM. The highest CSM was observed in age 80+ (HR = 5.037, 95% CI 2.606–9.736, *p* < 0.001) and patients with distant metastases (HR = 1.953, 95% CI 1.396–2.733, *p* < 0.001). Patients with malignant fibrous histiocytomas (HR = 0.572, 95% CI 0.378–0.865, *p* = 0.008) and those who underwent surgery (HR = 0.581, 95% CI 0.436–0.774, *p* < 0.001) had a lower CSM.

Table 3 summarizes the results of multivariate cox proportional hazard regression analyses of characteristics influencing OM and CSM of patients with PCS diagnosed between 2000 and 2017. The following groups were found to have higher overall mortality: age 80+ (HR = 13.261, 95% CI 5.839–30.119, *p* < 0.001), followed by age 60–79 (HR = 1.916, 95% CI 1.213–3.025, *p* = 0.005); and advanced disease with distant metastasis (HR = 2.013, 95% CI 1.355–2.99, *p* = 0.001), followed by regional involvement (HR = 1.518, 95% CI 1.041–2.214, *p* = 0.03). Lower OM rate was observed in patients with rhabdomyosarcoma (HR = 0.364, 95% CI 0.154–0.86, *p* = 0.021) and widowed (HR = 0.506, 95% CI 0.263–0.977, *p* = 0.042). Age 80+ (HR = 11.177, 95% CI 4.449–28.08, *p* < 0.001) and advanced disease with distant metastases (HR = 2.117, 95% CI 1.37–3.271, *p* = 0.001) have the highest CSM. Patients with rhabdomyosarcoma (HR = 0.344, 95% CI 0.128–0.929, *p* = 0.035) have a lower CSM. 

**Figure 1:**
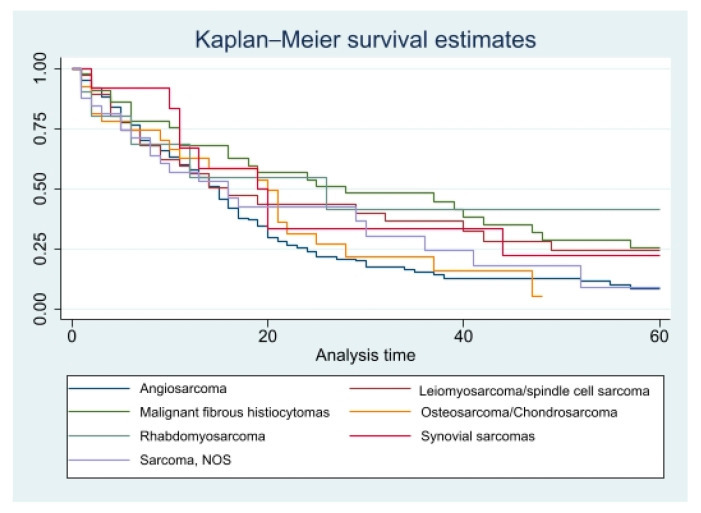
PCS specific 5-year survival for all patients based on Histopathology. Pr > chi2 = 0.0572.

**Figure 2:**
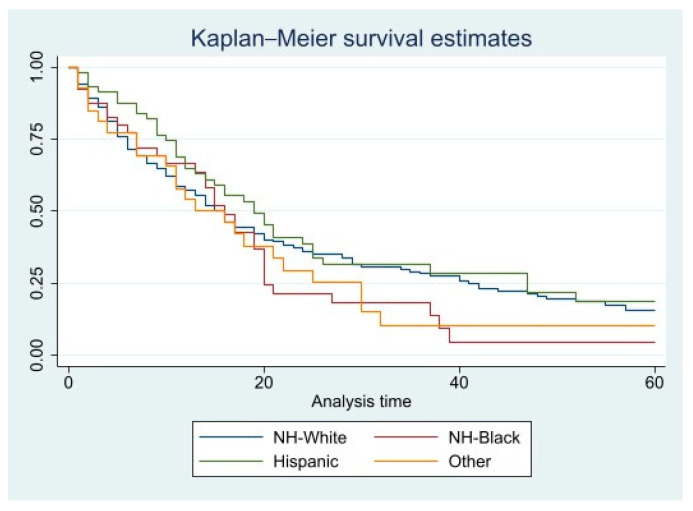
PCS specific 5-year survival for all patients based on race. Pr > chi2 = 0.2949.

**Figure 3:**
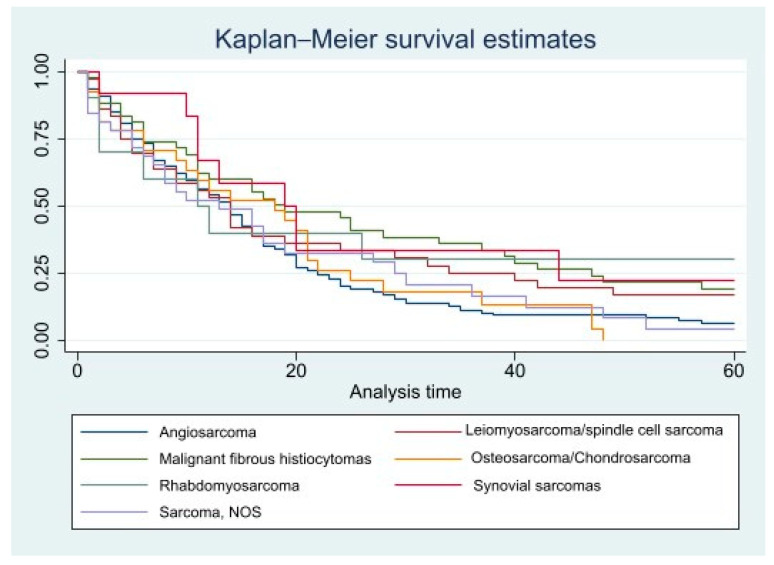
Overall, 5-year survival for all patients is based on Histopathology. Pr > chi2 = 0.1052.

**Figure 4:**
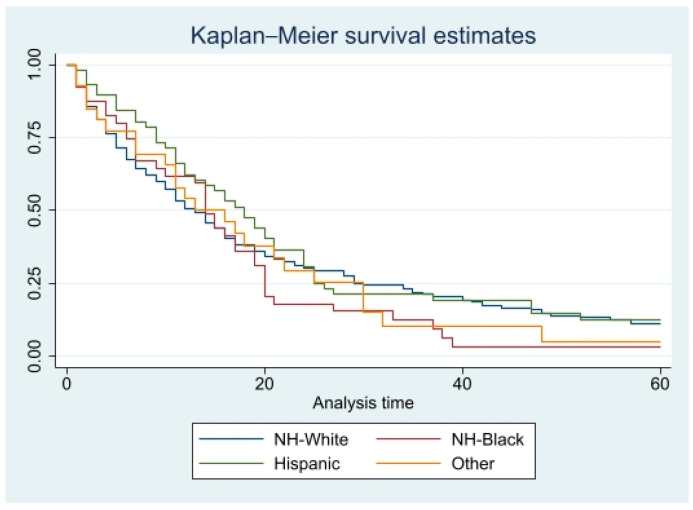
Overall, 5-year survival for all patients is based on race. Pr > chi2 = 0.4172.

Kaplan–Meier curves for five-year crude survival estimate of all the variables were generated. However, there were no statistically significant *p* values for any of these variables. CSS curves for histopathology and races can be found in Figure 1 and Figure 2, and OS curves for the same variables can be found in Figure 3 and Figure 4. 

## 4. Discussion

Primary cardiac sarcomas are rare. In this United States-based population cohort using the SEER database, we found that age at diagnosis and advanced disease with metastasis are associated with poor prognosis. Surgical intervention of the primary tumor was associated with lower mortality in the crude analysis but did not make any difference in the multivariate analysis. Patients with malignant fibrous histiocytoma had the best prognosis in the crude analysis but not in the multivariate analysis, whereas rhabdomyosarcoma had the best prognosis in the adjusted multivariate analysis. Angiosarcoma was the most encountered histologic subtype.

Prior literature has demonstrated that rhabdomyosarcomas make up approximately 20% of malignant sarcomas of the heart and are usually multifocal [14]. Our study had different findings, with rhabdomyosarcoma representing only 3.31% of our cohort. Sarcomatous tumors of the heart proliferate rapidly causing myocardial replacement and can lead to widespread metastatic disease [14]. Angiosarcomas, the most common subtype in adults, are aggressive and rapidly invading adjacent structures, and 47% to 89% of patients present with lung, liver, or brain metastases by the time of diagnosis [15,16]. This rapid proliferation and high representation of angiosarcoma might be the reason most patients in our cohort were diagnosed at advanced disease stages with distant metastases. 

Most of our patients were diagnosed between 40 and 59 years old, which differs from the findings in the study by Yin et al. where patients were mostly diagnosed between 20 and 40 years old [12]. However, a similar trend for seen in the study by Hammami et al. where patients were diagnosed between 31 and 60 years old [17]. The majority of patients in our cohort were white, representing more than half of the population in this study, which is in adequacy with the literature [12,17]. This trend can be explained by the disparity in healthcare access between the races [18]. 

Patients living in crowded areas and those with a higher annual revenue were most likely to be diagnosed with PCS in our cohort. Metropolitans with a crowded population tend to be better served medically with higher accessibility to advanced imaging techniques and better expertise. Likewise, patients with higher revenue are more likely to have better insurance coverage and means of affording other out-of-pocket medical expenses, thus increasing their diagnosis yield. Previous observational studies carried out on cancer patients have found a better outcome in married patients compared to their nonmarried counterparts in terms of OM and CSM [19,20,21,22,23,24,25,26,27,28,29]. Better spousal and social supports were possible explanations for better survival. However, that observation did not hold true in our cohort. Furthermore, widowed patients had a better OM compared to other marital statuses. However, this could be due to the relatively small number of widowed patients in our cohort. 

Surgical resection is the mainstay of treatment for PCS. Despite the overall poor survival, patients who do undergo complete resection have better outcomes compared with those who undergo incomplete resection. The roles of radiation, chemotherapy, cardiomyopathy, and cardiac transplantation, including auto-transplantation, are still controversial due to the rarity of the disease, lack of standardized therapies, and scarcity of data in the literature [30]. The study by Yin et al. found that chemotherapy and surgical resection were associated with better prognosis [12]. Half of the patients in our cohort underwent chemotherapy and more than half underwent surgical resection of the primary tumor. Our study revealed a better prognosis for patients with surgical resection in the crude analysis. However, these results were not sustained in the adjusted multivariate analysis. Neither surgical resection nor chemotherapy were associated with better prognosis in our cohort. 

As stated above, complete resection is the treatment of choice for PCS even though most patients develop recurrent disease that is fatal despite complete resection. The study by Centofanti et al., that enrolled patients from 1980 to 1997, found that effective palliation is possible with resection of malignant tumors and effective adjuvant therapy may improve prognosis [30]. The study by Bakaeen et al. [31], that enrolled patients from 1975 to 2002, revealed that there was good local tumor control with surgery, but no effect was found on the mortality given the metastatic nature of the PCS at the time of surgery. Ramlawi et al., enrolling patients from 1990 to 2015 in their single institution study, found a one year post-operative mortality rate of 35% [32]. Kosuga et al., that carried out a study between 1978 and 1999, found a poor prognosis of malignant PCS if only debulked but a great benefit of aggressive surgery if carried out with palliative intentions until adjuvant therapy is available [33]. The study by Raaf et al. published in 1994 concluded that surgical resection, adjuvant chemotherapy, and radiation therapy can also cause symptoms and prolong survival [34]. 

As evidenced by the above-mentioned studies over time, surgical resection remains the mainstay of treatment in patients with PCS. There are currently contrasting and mixed data showing the benefits of adjuvant chemotherapy or radiation in the survival of patients with PCS. As stated earlier, in our cohort, when adjusting for covariates to avoid confounding factors, surgery of primary, adjuvant chemotherapy, and radiation did not influence mortality. Therefore, we propose that those treatment modalities should only be reserved for palliative purposes and in an effort to improve the patient’s quality of life. We hope to encourage clinicians to avoid exposing patients with PCS, especially those with advanced age and or disease, to side effects of radiation, chemotherapy, and surgery if those are not done with the intention to alleviate the patient’s suffering. In the era of emergence of immunotherapy in the treatment of refractory solid tumors, particularly Chimeric Antigen Receptor (CAR) T Cell therapy, promising data have emerged in the treatment of sarcomas [35]. 

Certain limitations must be considered when interpreting the results of this study. Our study was mainly carried out on PCS, this makes it difficult to generalize our results to metastatic cardiac sarcomas. Information gathered on patients that underwent surgery was not complete. We were unable to find out if patients underwent complete or incomplete resection. Furthermore, the SEER database publicly available does not provide information on comorbidities. Information provided about patients’ living areas is limited to states and information about specific areas of origin is not provided in the database. However, this study has the merit of collecting data from the largest cancer database in the USA. We were also able to enroll an adequate sample size despite the rarity of the pathology. 

## 5. Conclusions

Data on PCS are very pauce in the literature mainly due to the scarcity of pathology. A dismal prognosis is associated with PCS given its aggressive behavior. As one could expect, advanced age and advanced disease with metastasis are independent factors of poor prognosis, as evidenced in our cohort over the past two decades. No positive prognostic value was observed after resecting the primary tumor. Rhabdomyosarcoma was the histologic type with the best prognosis. Furthermore, adjuvant chemotherapy and radiation did not affect mortality. Given the aggressive nature and dismal prognosis of PCS, treatment of such patients should mainly focus on improving the quality of life. Clinicians should weigh the pros and cons of proceeding with such therapies as the side effects might worsen the patient’s quality of life without prolonging survival. We propose that, for the elderly and those with advanced disease, the above-mentioned therapies should only be carried out with the intention of palliation. With newer and promising data on CAR T cell therapy on solid tumors, including sarcomas, we hope that more clinical trials involving patients with PCS will be carried out, as the need to find new treatment modalities for this dismal pathology is urging. 

## Figures and Tables

**Table 1:** Demographic and Clinicopathologic characteristics of US patients with Primary cardiac sarcoma between 2000 and 2017.

**Characteristics**		
	**N *=***	%
**Total**	362	100
**Gender**		
Female	178	49.17
Male	184	50.83
**Age at diagnosis, y.o**		
0–39	116	32.04
40–59	137	37.85
60–79	86	23.76
80+	23	6.35
**Race**		
Non-Hispanic white	213	58.84
Non-Hispanic black	44	12.15
Hispanic	68	18.78
Other	37	10.22
**Histopathology**		
Angiosarcoma	172	42.51
Rhabdomyosarcoma	12	3.31
Malignant fibrous histiocytomas	51	14.09
Leiomyosarcomas/spindle cell sarcomas	42	11.60
Sarcoma, NOS	38	10.50
Synovial sarcomas	14	3.87
Osteosarcoma/Chondrosarcoma	33	9.12
**Tumor stage**		
Localized	96	26.52
Regional	110	30.39
Distant	140	38.67
Unknown	16	4.42
**Living area**		
Counties in metropolitan areas of 1 million persons	215	59.39
Counties in metropolitan areas of 250,000 to 1 million persons	79	21.82
Counties in metropolitan areas of 250,000 persons	27	7.46
Nonmetropolitan counties adjacent to a metropolitan area	15	4.14
Nonmetropolitan counties not adjacent to a metropolitan area	26	7.18
**Income per year**		
$ <$45,000	18	4.97
$45,000–54,999	57	15.75
$55,000–64,999	81	22.38
$65,000–74,999	90	24.86
$75,000+	116	32.04
**Marital Status**		
Married	197	54.42
Single	97	26.80
Divorced/separated	33	9.12
Widowed	21	5.80
Unknown	14	3.87
**Chemotherapy**		
No	181	50.00
Yes	181	50.00
**Radiation**		
No	285	79.83
Yes	72	20.17
**Surgery**		
No	123	33.98
Yes	237	65.47
Unknown	2	0.55
**Year of diagnosis**		
2000	17	4.70
2001	20	5.52
2002	13	3.59
2003	13	3.59
2004	26	7.18
2005	11	3.04
2006	13	3.59
2007	23	6.35
2008	9	2.49
2009	32	8.84
2010	22	6.08
2011	28	7.73
2012	14	3.87
2013	20	5.52
2014	28	7.73
2015	20	5.52
2016	23	6.35
2017	30	8.29

**Table 2:** Crude analysis of factors associated with all-cause mortality and Primary Cardiac Sarcoma related mortality among US patients between 2000 and 2017.

Characteristics	Overall Mortality. Crude Proportional Hazard Ratio (95% Confidence Interval)	Primary Cardiac Sarcoma Mortality. Crude Proportional Hazard Ratio (95% Confidence Interval)
**Gender**		
Female	1.00 (reference)	1.00 (reference)
Male	0.964 (0.758–1.226)	0.995 (0.766–1.293)
**Age at diagnosis, y.o**		
0–39	1.00 (reference)	1.00 (reference)
40–59	1.212 (0.911–1.615)	1.258 (0.925–1.712)
60–79	1.429 (1.028–1.986) **	1.35 (0.939–1.941)
80+	5.958 (3.357–10.575) ***	5.037 (2.606–9.736) ***
**Race**		
Non-Hispanic white	1.00 (reference)	1.00 (reference)
Non-Hispanic black	1.222 (0.851–1.754)	1.257 (0.851–1.857)
Hispanic	0.833 (0.602–1.154)	0.827 (0.578–1.182)
Other	1.063 (0.685–1.648)	1.212 (0.77–1.906)
**Histopathology**		
Angiosarcoma	1.00 (reference)	1.00 (reference)
Rhabdomyosarcoma	0.608 (0.283–1.307)	0.501 (0.204–1.231)
Malignant fibrous histiocytomas	0.657 (0.455–0.95) **	0.572 (0.378–0.865) ***
Leiomyosarcomas/spindle cell sarcomas	0.787 (0.535–1.16)	0.734 (0.48–1.123)
Sarcoma, NOS	0.965 (0.64–1.456)	0.853 (0.539–1.349)
Synovial sarcomas	0.535 (0.271–1.056)	0.608 (0.307–1.203)
Osteosarcoma/Chondrosarcoma	0.987 (0.646–1.507)	0.932 (0.589–1.474)
**Tumor stage**		
Localized	1.00 (reference)	1.00 (reference)
Regional	1.334 (0.964–1.846)	1.386 (0.972–1.976)
Distant	1.888 (1.389–2.566) ***	1.953 (1.396–2.733) ***
**Living area**		
Counties in metropolitan areas of 1 million persons	1.00 (reference)	1.00 (reference)
Counties in metropolitan areas of 250,000 to 1 million persons	1.01 (0.747–1.366)	0.993 (0.714–1.381)
Counties in metropolitan areas of 250,000 persons	1.037 (0.662–1.623)	1.051 (0.648–1.703)
Nonmetropolitan counties adjacent to a metropolitan area	1.666 (0.923–3.007)	1.614 (0.846–3.081)
Nonmetropolitan counties not adjacent to a metropolitan area	1.128 (0.721–1.766)	1.16 (0.716–1.88)
**Income per year**		
$ <$45,000	1.00 (reference)	1.00 (reference)
$45,000–54,999	0.72 (0.372–1.392)	0.842 (0.392–1.805)
$55,000–64,999	0.689 (0.363–1.309)	0.774 (0.367–1.632)
$65,000–74,999	0.601 (0.316–1.144)	0.729 (0.346–1.534)
$75,000+	0.624 (0.332–1.172)	0.755 (0.363–1.569)
**Marital Status**		
Married	1.00 (reference)	1.00 (reference)
Single	0.854 (0.643–1.135)	0.783 (0.572–1.071)
Divorced/separated	1.121 (0.728–1.726)	1.121 (0.707–1.778)
Widowed	1.203 (0.717–2.019)	1.207 (0.694–2.099)
**Chemotherapy**		
No	1.00 (reference)	1.00 (reference)
Yes	0.928 (0.724–1.19)	0.956 (0.729–1.253)
**Radiation**		
No	1.00 (reference)	1.00 (reference)
Yes	0.931 (0.7–1.24)	0.937 (0.688–1.278)
**Surgery**		
No	1.00 (reference)	1.00 (reference)
Yes	0.606 (0.465–0.791) ***	0.581 (0.436–0.774) ***

*** *p* < 0.01, ** *p* < 0.05.

**Table 3:** Multivariate cox proportional hazard regression analyses of factors affecting all-cause mortality and Primary Cardiac Sarcoma related mortality among US patients between 2000 and 2017.

Characteristics	Overall Mortality. Adjusted Proportional Hazard Ratio (95% Confidence Interval)	Primary Cardiac Sarcoma Mortality. Adjusted Proportional Hazard Ratio (95% Confidence Interval)
**Gender**		
Female	1.00 (reference)	1.00 (reference)
Male	0.952 (0.692–1.309)	0.917 (0.649–1.296)
**Age at diagnosis, y.o**		
0–39	1.00 (reference)	1.00 (reference)
40–59	1.201 (0.847–1.703)	1.173 (0.81–1.699)
60–79	1.916 (1.213–3.025) ***	1.61 (0.976–2.657)
80+	13.261 (5.839–30.119) ***	11.177 (4.449–28.08) ***
**Race**		
Non-Hispanic white	1.00 (reference)	1.00 (reference)
Non-Hispanic black	1.446 (0.919–2.276)	1.557 (0.962–2.52)
Hispanic	1.051 (0.691–1.597)	1.015 (0.645–1.596)
Other	1.174 (0.709–1.944)	1.379 (0.814–2.336)
**Histopathology**		
Angiosarcoma	1.00 (reference)	1.00 (reference)
Rhabdomyosarcoma	0.364 (0.154–0.86) **	0.344 (0.128–0.929) **
Malignant fibrous histiocytomas	0.668 (0.422–1.056)	0.62 (0.373–1.029)
Leiomyosarcomas/spindle cell sarcomas	0.751 (0.458–1.229)	0.705 (0.411–1.207)
Sarcoma, NOS	1.376 (0.818–2.314)	1.322 (0.75–2.33)
Synovial sarcomas	0.603 (0.271–1.341)	0.738 (0.329–1.657)
Osteosarcoma/Chondrosarcoma	1.355 (0.792–2.316)	1.37 (0.758–2.476)
**Tumor stage**		
Localized	1.00 (reference)	1.00 (reference)
Regional	1.518 (1.041–2.214) **	1.574 (1.042–2.379) **
Distant	2.013 (1.355–2.99) ***	2.117 (1.37–3.271) ***
**Living area**		
Counties in metropolitan areas of 1 million persons	1.00 (reference)	1.00 (reference)
Counties in metropolitan areas of 250,000 to 1 million persons	0.999 (0.692–1.44)	0.927 (0.624–1.377)
Counties in metropolitan areas of 250,000 persons	0.654 (0.372–1.148)	0.586 (0.318–1.082)
Nonmetropolitan counties adjacent to a metropolitan area	1.16 (0.556–2.421)	1.007 (0.444–2.283)
Nonmetropolitan counties not adjacent to a metropolitan area	1.226 (0.663–2.268)	1.434 (0.743–2.771)
**Income per year**		
$ <$45,000	1.00 (reference)	1.00 (reference)
$45,000–54,999	1.049 (0.48–2.292)	1.531 (0.62–3.781)
$55,000–64,999	0.856 (0.379–1.93)	1.207 (0.475–3.069)
$65,000–74,999	0.778 (0.339–1.787)	1.158 (0.448–2.991)
$75,000+	0.812 (0.353–1.864)	1.199 (0.463–3.104)
**Marital Status**		
Married	1.00 (reference)	1.00 (reference)
Single	0.834 (0.573–1.215)	0.681 (0.451–1.028)
Divorced/separated	1.27 (0.746–2.163)	1.183 (0.664–2.108)
Widowed	0.506 (0.263–0.977) **	0.576 (0.282–1.18)
**Chemotherapy**		
No	1.00 (reference)	1.00 (reference)
Yes	0.97 (0.704–1.337)	1.047 (0.739–1.484)
**Radiation**		
No	1.00 (reference)	1.00 (reference)
Yes	0.94 (0.674–1.313)	0.829 (0.578–1.188)
**Surgery**		
No	1.00 (reference)	1.00 (reference)
Yes	0.735 (0.508–1.062)	0.724 (0.489–1.072)

*** *p* < 0.01, ** *p* < 0.05.

## Data Availability

The data used and/or analyzed in this study are available in the Surveillance, Epidemiology, and End Results (SEER) Database of the National Cancer Institute (http://seer.cancer.gov accessed on 27 July 2021).
